# Forever chemicals: the persistent effects of perfluoroalkyl and polyfluoroalkyl substances on human health

**DOI:** 10.1016/j.ebiom.2023.104806

**Published:** 2023-09-13

**Authors:** eBioMedicine

Perfluoroalkyl and polyfluoroalkyl substances (PFAS) are a diverse group of thousands of humanmade chemical pollutants that have the potential to persist in both the environment and the human body. An increasing number of epidemiological studies have documented adverse health risks that are associated with PFAS exposure, including increased risks of some cancers, reduced immune function, and developmental delays in children. A systematic review published in *Exposure and Health* in 2022 reported that the cost of PFAS-attributable diseases in the USA is at least US$5.5 billion annually and could be as high as $62 billion annually. The European Chemicals Agency estimates that 4.4 million tonnes of PFAS will be released into the environment by 2053 if no urgent action is taken to restrict their use.

The chemical properties of PFAS make them desirable and profitable for use in industry (eg, firefighting foams) and consumer products (eg, water-resistant clothing, food containers, and non-stick cooking utensils). Their use at industrial, military, and manufacturing sites is an occupational health risk. For example, a nested case-control study of men in the US air force published in July, 2023, in *Environmental Health Perspectives* reported a positive association between increased serum concentrations of some PFAS chemicals and the risk of testicular germ cell tumours. These sites are also a predominant source of pollution into the surrounding environment.

Leaching from contaminated sites into waterways has made drinking water a substantial route of PFAS exposure for many people. In March, 2023, the US Environmental Protection Agency proposed strict new standards to reduce the amounts of six PFAS chemicals (ie, perfluorooctanoic acid [PFOA], perfluorooctanesulfonic acid [PFOS], perfluorononanoic acid [PFNA], perfluorohexane sulfonic acid [PFHxS], perfluorobutane sulfonate, and hexafluoropropylene oxide dimer acid) in drinking water in the USA. According to a study published in August, 2023, in *Environment International*, temporal sampling of 716 tap-water locations in the USA estimated that at least one PFAS could be detected in approximately 45% of drinking-water samples. Similar patterns of PFAS occurrence in drinking water have been reported in other countries (eg, Sweden, Czech Republic, China, and The Gambia).

Even in populations that are geographically distant from industrial sources of PFAS, exposure occurs through atmospheric and marine transport. Subsequent bioaccumulation up marine food chains means that people who rely on such diets are at risk of consuming dangerous amounts of PFAS. A July, 2023, a mixed-methods study in *The Lancet Planetary Health* assessed long-term exposure to PFAS in Inuit adults who consumed a traditional diet of polar-bear meat and ringed-seal meat. Measurements of whole-blood serum concentrations of PFAS and modelling estimated that 86% of the Ittoqqortoormiit Inuit population exceeded the European Food Safety Authority estimate for tolerable PFAS intake per week (ie, 4.4 ng per kg of bodyweight), putting them in the severe-risk category for immune suppression.

Possible immunotoxic effects of PFAS exposure have been highlighted in multiple studies. In a Danish biobank study published in December, 2020, in *PloS One*, perfluorobutanoic acid (PFBA; a short-chain PFAS that is known to accumulate in the lungs) was associated with increased severity of COVID-19. In a 2023 *Chemosphere* study, the impact on immune cell activation of PFBA plus two other short-chain PFAS (PFHxA, PFHxS) and three long-chain PFAS (PFOA, PFOS and PFNA) was evaluated. A significant decrease in all activation markers expressed by CD4+ and mucosal-associated invariant T cells was observed after pre-exposure of human peripheral blood mononuclear cells to PFAS mixtures in the concentrations found in human serum. PFOA and PFOS, either alone or combined, led to inflammasome priming and activation in human bronchial epithelial cells in a study published in May, 2023, in the *International Journal of Molecular Sciences*. In the same study, RNA sequencing and gene-set enrichment analysis of lungs from mice exposed to PFOA in their drinking water showed upregulation of cytokines and immunity-related genes.

Of particular concern is the diminished immunisation efficiency observed in children exposed to PFOA. In 237 infants in a randomised controlled trial conducted in Guinea-Bissau in 2020, serum PFAS and measles-antibody concentrations were measured. The trial, published in *Environmental Health Perspectives*, reported that increased PFOS and PFDA was associated with reduced measles-antibody concentrations at the 9-month visit. A January, 2022, cross-sectional study of children aged 7–12 years in Greenland, published in *Environmental Research*, reported an association between childhood PFAS exposure and decreased concentrations of diphtheria-vaccine antibodies and tetanus-vaccine antibodies. Exposure to all seven PFAS that were measured was associated with an increased likelihood of insufficient diphtheria antibodies for protection against infection.

Young children and pregnant people were recognised as particularly susceptible to PFAS-associated health effects in comprehensive guidance released by the US National Academies of Sciences, Engineering, and Medicine. In 2022, the US Environmental Working Group reviewed 40 studies that detected PFAS in cord blood and reported health outcomes. More than a third of these studies found an association between PFAS detection in cord blood, the presence of PFAS later in childhood, and increased health risks. Prospective birth cohorts are valuable resources in efforts to document pregnancy and health outcomes. In a cross-sectional study of 72 pregnant people from the Generation C birth cohort, a negative correlation between SARS-CoV-2 anti-spike IgG antibody levels and 16 measured PFAS was reported. In August, 2023, in *eBioMedicine*, results from the COPSAC2010 birth cohort were published. Increased PFOS and PFOA exposure during pregnancy was associated with a non-atopic asthma phenotype in the child by age 6 years. This association was primarily driven by prenatal exposure to PFAS. High serum concentrations of PFOA and PFHxS were associated with an increased risk of preterm birth in a *Nature Communications* study that used data from the Atlanta African American Maternal-Child Cohort. Metabolomics was conducted on newborn dried-blood spots, revealing an association between reduced gestation and eight metabolomic pathways and indicating altered neuroendocrine function and bioenergetic processes. Understanding whether, and to what extent, these alterations persist beyond birth, as well as any subsequent health effects, is important.

Many studies to date have focused on so-called legacy PFAS, primarily PFOS and PFOA, which are already restricted internationally under the Stockholm Convention on Persistent Organic Pollutants. However, thousands of PFAS and their relative combinations and doses are yet to be explored and current restrictions on PFAS production and use are governed at the individual-chemical level. In early 2023, the European Chemicals Agency announced an ambitious proposal to restrict the production and use of approximately 10,000 PFAS. However, because of the long half-lives of PFAS that are already in the environment, research into PFAS-attributable disease must continue. At *eBioMedicine*, we seek high-quality toxicological and mechanistic studies to reveal the complex interactions between PFAS and adverse health outcomes and to support the ban of harmful chemicals.

